# 

**Published:** 1874-11

**Authors:** 


### Table of Contents.

### ORIGINAL COMMUNICATIONS.

Case of Occlusion of the Os-Uteri at Term, Resulting in Delivery through the Rectum. *By Goronwy Owen, M.D* .................. 449
Chloroform in Placenta Prævia and Post-Partum Hemorrhage. *By P. S. Verdery, M.D., Chapel, Hill, Georgia* .................. 454
Lacto-Peptine. *By Laurence Alexander, M.D., Yorkville, S. C.* .................. 456
Delivery Without Rupture of Hymen--Remarks. *By J. A. Meek, M.D., Jonesboro, Ark.* .................. 457

### ATLANTA ACADEMY OF MEDICINE.

Case of Sudden and Fatal Coma—Treatment of Coma—Peculiar Exanthem in Cobb county—Correlation between Diphtheria and Scarlatina—Malarial Hæmaturia—Ergot in Enlarged Prostate—Carbolic Acid in Hemorrhoids—Imperforate Anus, with entire absence of Rectum—Pelvic Abscess, discharged through the Colon .................. 458

### OUR EXCHANGES.

Medical Ethics .................. 463
Chloroform Narcosis .................. 464
Blood Drinkers—Chloroform in Strychnia-Poisoning .................. 465
Belladonna in Spasmodic Asthma .................. 466
Dyspepsia .................. 467
Sexual Influence of Anæsthetics .................. 468
Female Diseases from Stair-Climbing .................. 469
Injection of Pulmonary Cavities .................. 470
Eight Months' Constipation .................. 472
Vaginal Injections of Hot Water .................. 473
Relation of Ozone to Disease .................. 474
Hernia Reduced by Distension—Dispersion of Tumors by Puncture .................. 475
Guarana in Headache .................. 476
Washing out the Stomach .................. 477
Ovarian Physiology .................. 478
Ethics—Obstinate Vomiting—Cancer .................. 479
Prescriptions—Quinia Pills—Chronic Gonorrhœa .................. 480
Modern Isms and Ologies, (poetical) .................. 481
Francis Anstie .................. 482
Manipulation in Stricture—Alaska Candles .................. 483
Chorea—Gossypium in Dysmenorrhœa .................. 484
Ether and Chloroform—effect on Fœtus .................. 485
Hypodermic Chloroform—Intestinal Invagination .................. 486
To Check Cough—Ergot in Dysentery .................. 487
Chloroformization—Eczema Capitis .................. 488
Explosion Pot. Chlor.—Chloral in Trismus .................. 489
Success in Medical Practice .................. 490
English Opinion of American Medical Association .................. 491
Castor Oil Injection Vomited—Tape Worms .................. 492
Treatment of Impetigo—Warning to Ladies .................. 493
Tape Worm—Camphor Chloral in Neuralgia .................. 494
New Property of Albumen—Low Temperatures .................. 495
Common Glue Dressing—Ether in Labor .................. 496
Chloral in Cancer—Bromide of Camphor .................. 497

### BIBLIOGRAPHICAL.

Thomas on Diseases of Women .................. 498
Hartshorne—Essentials of Practice .................. 502
Clay—Hand-book of Obstetric Surgery .................. 503
Swain—Surgical Emergencies .................. 505
Cohen—Croup and Tracheotomy .................. 506

### EDITORIAL.

Salutatory—Valedictory .................. 508
Ethics—Dr. Hammond and the *Record* .................. 509
The Hot Vaginal Douche .................. 512

